# *In vivo* tau staging in Alzheimer’s disease

**DOI:** 10.18632/aging.204293

**Published:** 2022-09-14

**Authors:** Joseph Therriault, Serge Gauthier, Pedro Rosa-Neto

**Affiliations:** 1 Translational Neuroimaging Laboratory, The McGill University Research Centre for Studies in Aging, Douglas Hospital, McGill University, Montreal, Canada; 2 Department of Neurology and Neurosurgery, McGill University, Montreal, Canada; 3 Montreal Neurological Institute, Montreal, Canada

**Keywords:** disease staging, Alzheimer’s disease, amyloid-β, tau, biomarkers

Disease staging systems allow for the measurement of disease severity based on the identification of important milestones in the natural history of a disease. Staging systems can be used to help guide clinical care, serve as inclusion criteria for therapeutic trials, and furthermore provide a consistent framework for interpreting findings from different studies in different populations. Disease staging systems can also be used to model the progression of clinical and biomarker changes in relation to important disease landmarks. In Alzheimer’s disease (AD), which is defined by the presence of amyloid-β plaques and tau neurofibrillary tangles [1,2], staging systems have been devised based on the anatomical distribution of neuropathology at autopsy. Staging of AD is already conducted routinely: post-mortem neuropathological assessments of AD, considered the gold standard in AD diagnosis, rely on histopathological staging systems [1].

**Figure 1:**
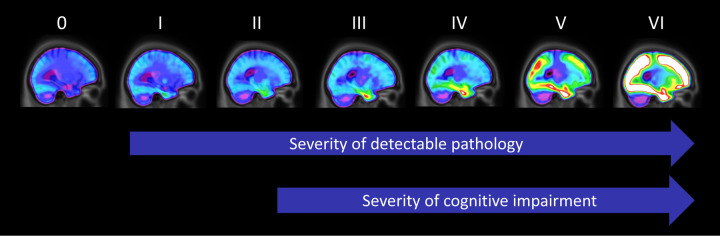
Progression of PET-based Braak stages .

Although most *in vivo* research has focused on dichotomous classification of amyloid-PET and tau-PET imaging into +/- groups, the spatial resolution of PET provides an opportunity for staging based on the anatomical distribution of pathology, similar to post-mortem staging systems [3]. Due to established relationships between tau, neurodegeneration and cognitive dysfunction, the Braak neurofibrillary tangle staging system [4] provides a useful framework for staging tau pathology, as well as AD severity. Recent work from our group has applied the Braak staging system to [^18^F]MK6240 tau-PET data [5]. After assigning a Braak stage to all subjects based on the topography of tau-PET abnormality (Figure 1), individual-level Braak stages were employed to model the trajectory of other AD biomarkers, as well as disease symptoms. Overall, we observed that most fluid p-tau biomarkers became subtly elevated at Braak stage II. At stages III and IV, this abnormality increased in magnitude, and subsequently plateaued at stages V and VI. Very subtle memory decline could be identified by stage II, with mild cognitive symptoms detectable by stages III and VI. Braak stages V and VI were only observed in individuals with overt cognitive impairment (mild cognitive impairment or dementia). A unique feature of *in vivo* disease staging, as opposed to neuropathology, is the ability to track the evolution of disease in the same individual over time. Using longitudinal imaging, we observed that amyloid-PET abnormality at baseline was associated with different rates of Braak stage progression. Progression from early Braak stages (0-II) took place in the presence or absence of abnormal amyloid. However, progression from Braak stage III and above during two-year follow-up occurred exclusively in the presence of abnormal amyloid-β.

Taken together, these results showcase how applying disease staging systems to living humans can provide new insights into disease progression [3]. Another important advantage of *in vivo* disease staging is the ability to monitor the severity of AD specifically. This is in contrast to clinical dysfunction, which is often the result of multiple neuropathological processes. Furthermore, cognitive reserve, which can influence the extent to which individuals can carry brain pathology without symptoms, also complicates clinical staging. PET-based Braak staging can also identify preclinical disease (detectable tau pathology in cognitively unimpaired individuals). Therefore, *in vivo* staging of Alzheimer’s disease has greater sensitivity and specificity over clinical assessment of symptoms [3].

PET-based Braak staging may also prove useful for clinical trial enrolment. Identifying individuals at a specific stage of tau abnormality will allow for a more homogeneous study population, potentially reducing the variance in longitudinal cognitive decline. Furthermore, studies aimed at halting the progression of tau pathology may seek to recruit patients at a specific early stage. Finally, tau-PET can be used to monitor disease progression in clinical trials, with lack of tau progression as a possible signal of effectiveness. This is similar to the recent phase II donanemab trials which recruited amyloid-positive individuals with intermediate levels of tau-PET uptake, which met primary endpoints in phase II [6].

The recent approval of aducanumab (Aduhelm) highlights the need for biomarkers to assess the severity of AD [7]. If evidence supports the optimal utility of anti-amyloid therapies (such as aducanumab or other monoclonal antibodies) for specific stages of AD, biomarker-driven staging systems may be well-positioned to identify who may optimally respond to a specific therapeutic option. Furthermore, if and when other therapeutic options are available, biomarker-driver staging systems will be able to determine the optimal therapeutic window for specific therapeutic modalities (i.e. anti-amyloid, anti-tau, combination therapies).

Several unanswered questions remain. The first is the degree of correspondence between PET-based Braak stage and Braak stage at autopsy. Because of limitations of PET’s sensitivity, some discordance between antemortem and postmortem Braak stage is expected. Furthermore, it must be highlighted that the methodology of *in vivo* PET staging and *ex vivo* neuropathological staging are very different: *in vivo* PET-based Braak staging relies on surpassing predefined thresholds of abnormality with specific brain ROIs representing specific Braak stages. In contrast, histopathological Braak staging relies on the detection of neurofibrillary tangles using staining techniques. Therefore, identification of even small numbers of neurofibrillary tangles using histopathological techniques can place an individual at a more advanced Braak stage, as a higher concentration of neurofibrillary tangles would be needed for the same individual to exceed a predefined threshold for abnormality. Finally, it is important to emphasize that staging systems involve dimensionality reduction. Despite the fact that atypical clinical variants of AD largely conform to the Braak system, some information about the spatial distribution of tau in atypical AD [8] is lost when only considering an individual’s tau stage.
